# Impact of Dehydroepiandrosterone (DHEA) on Bone Mineral Density and Bone Mineral Content in a Rat Model of Male Hypogonadism

**DOI:** 10.3390/vetsci7040185

**Published:** 2020-11-23

**Authors:** Hussein F. Sakr, Abdelaziz M. Hussein, Elsayed A. Eid, Ammar Boudaka, Lashin S. Lashin

**Affiliations:** 1 Department of Physiology, College of Medicine and Health sciences, Sultan Qaboos University, Muscat 123, Oman; sakr_doctor@yahoo.com (H.F.S.); boudaka@squ.edu.om (A.B.); 2 Medical Physiology Department, Faculty of Medicine, Mansoura, University, Mansoura 35516, Egypt; lashin.saad@yahoo.com; 3 Department of Endocrinology and Internal Medicine Department, Delta University for Science and Technology, Gamasa 35511, Egypt; elsayed.abdelfattah@deltauniv.edu.eg or; 4 Department of Medical Physiology, Horus University-Egypt, New Damietta 12613, Egypt

**Keywords:** DHEA, osteoporosis, OPG, TRAP-5B, RANK, osteocalcin, ALP

## Abstract

Objectives: The present study examined the effect DHEA (dehydroepiandrosterone) on bone mineral content (BMC) and bone mineral density (BMD) and biomarkers of bone remodeling in orchidectomized male rats. Material and Methods: A total of 32 male rats were divided equally into four groups (n = 8): (i) control group (C), (ii) control treated with DHEA (Control + DHEA), (iii) orchidectomized (ORCH) group that underwent bilateral orchidectomy and (iv) orchidectomized (ORCH) rats treated with DHEA (ORCH+DHEA). DHEA treatment started 4 weeks after orchidectomy and continued for 12 weeks. After 12 weeks the bone mineral density (BMD) and bone mineral content (BMC) were assayed in the tibia and femur of the right hind limb of each rat. We also measured the serum levels of the bone turnover markers deoxypyridinoline (Dpd), N-telopeptide of type I collagen (NTx), alkaline phosphatase (ALP), tartrate-resistant acid phosphatase 5b (TRAP-5b) and osteocalcin (OC) as well as receptor activator of nuclear factor kappa B (RANK) and osteoprotegerin (OPG). Results: Orchidectomy in rats caused significant reduction in BMD, BMC, serum levels of testosterone, PTH (parathyroid hormone), OPG, OC and ALP with significant rise in serum levels of TRAP-5B, RANK, Dpd and NTx1 (*p* < 0.05). On the other hand, DHEA therapy for 12 weeks caused significant improvement in all studied parameters except NTx1 (*p* < 0.05). Conclusions: DHEA corrected hypogonadism-induced osteoporosis in male rats probably via inhibiting osteoclastogenesis, stimulating the activity of osteoblasts and stimulating the secretion of PTH and testosterone.

## 1. Introduction

Bone is not a static but dynamic tissue that shows a continuous process of bone resorption and apposition (building up) or remodeling [1]. The process of bone remodeling plays a crucial role in keeping calcium plasma levels constant. In osteoporosis, the bone resorption is greater than bone apposition leading to loss of bone [2]. When osteoblasts are activated by inducers such as parathyroid hormone (PTH), interleukin 1 (IL-1), tumor necrosis factor (TNF) or prostaglandin (PGE-2), they secrete factors that activate the osteoclasts to resorb bone [2]. Bone mineral density (BMD) decreases in both sexes with advancing age at both cancellous and cortical sites [3]. Loss of bone mass in cancellous bone is accelerated after menopause in women, while in men it occurs later in life than in women [3] and increases progressively with advancing age, particularly after the age of 70 years [4]. Serum testosterone declines slowly in men with age, so a mild age-related hypogonadism is often frequent in older men. There is a clear relationship between decline in BMD and age-related hypogonadism which is demonstrated as an increased risk for hip fracture in hypogonadal aging men [5]. It has also been shown that patients with hip fractures have low serum testosterone levels [6]. Moreover, it has been shown that BMD is directly related to the amount of testosterone available [7,8]. 

The process of bone remodeling can be regulated by several signaling pathways including osteoprotegerin (OPG), receptor activator of NF-kB (RANK) and RANK ligand (RANKL) which are considered as the major factors involved in osteoclastogenesis. The main role of RANKL, a member of the tumor necrosis factor (TNF) family, is to inhibit apoptosis of osteoclasts and stimulate the differentiation and activation of these cells, while OPG directly inhibits the binding of RANKL with RANK [9]. During bone remodeling, several bone resorbing markers, such as C- or N-terminal telopeptide of type I collagen (CTX or NTX), tartrate-resistant acid phosphatase 5b (TRAP-5b), pyridinolines and bone forming cytokines such as procollagen type I N-terminal propeptide (PINP), alkaline phosphatase (ALP), bone specific alkaline phosphatase (BS-ALP), or osteocalcin (OC), are released. 

Dehydroepiandrosterone (DHEA) and DHEA-sulphate (S) are pre-hormones. Although DHEA-S constitutes a circulating stock, it is hydrophilic and only lipophilic DHEA can be transformed in peripheral tissues to more potent androgens and estrogens [10]. In an in vitro analysis, primary human osteoblasts showed aromatase activity converting DHEA to estrone [11,12]. Another in vitro study showed that DHEA inhibits apoptosis and promotes proliferation of rat osteoblasts through MAPK signaling pathways, independently from androgens and estrogens [12]. These findings support a positive effect of DHEA on bone through conversion to estrogens, but also independently from its hormonal end products. In a group of 120 post-menopausal women aged 51–99 years, lumbar spine BMD was related to DHEA-S but not to estradiol plasma levels [11]. DHEA levels have been positively related to BMD in men [13] and post-menopausal women [11]. We hypothesize that DHEA could indirectly inhibit human osteoclastic resorption through promoting osteoblastic viability and osteoprotegerin (OPG) production, which is mediated by the mitogen-activated protein kinase signal pathway involving phospho-ERK1/2 [14].

## 2. Materials and Methods

### 2.1. Animal Housing and Diet

Thirty-two male Wistar-Kyoto rats, 12–14-weeks-old, weighing 300 ± 25 g were provided and housed in the small animal house of SQU, Muscat, Oman. The atmospheric temperature was controlled at 23 ± 1 °C and light/dark cycle was a 12 h light:12 h dark. Rats had free access to food and tap water. All experimental procedures in this study were approved by the Medical Research Ethics Committee of SQU, Muscat, Oman (IG/MED/PHYS/18/02, 18 Feb, 2018, Ethical Committee, Dr. Yahya AI-Wahaibi, dean of Research) and were carried out according to the Guide for the Care and Use of Laboratory Animals.

### 2.2. Experimental Groups

After one week of acclimatization in the lab environment, the animals were randomly allocated into 4 equal groups as follows: (i) control (sham operated) group, normal rats treated with saline; (ii) control treated with DHEA (Control + DHEA), normal rats treated with DHEA pellets (50 mg, Innovative Research America (Sarasota, FL), Cat#, NX-999) implanted subcutaneously to release for 12 weeks; (iii) orchidectomized (ORCH) group that underwent bilateral orchidectomy; and (iv) orchidectomized (ORCH) treated with DHEA (ORCH+ DHEA) [15]. DHEA treatments started 4 weeks after orchidectomy and continued for 12 weeks. 

### 2.3. Orchidectomy Rat Model

All details of the experimental procedures of bilateral orchidectomy were done under sodium pentobarbital (12 mg/kg, intraperitoneally) according to Erben et al. [16].

### 2.4. Blood Samples Collections 

Blood samples were collected by cardiac puncture at the time of sacrifice in tubes without anticoagulant and left for 10 min. The tubes were centrifuged at 4000 r/min for 10 min to obtain serum. The serum was stored at −20 °C until biochemical analysis. 

### 2.5. Measurement of Bone Mineral Density (BMD) and Bone Mineral Content (BMC) of Right Tibia and Femur

After blood sampling, the right hind limb of all rats was dissected from the hip joint. By using LUNAR PIXI #50778 DEXA scan (Lunar PIXImus Corporation Headquarters 726 Heartland Trail Madison, WI 53717), the bone mineral density and bone mineral content were measured in the right femur and tibia. 

### 2.6. Biochemical Parameters

ELISA kits were used for measurement of the serum levels of testosterone hormone kit catalog number ab108666 (Cusabio), parathyroid hormone (PTH) (MBS2700368), osteoprotegerin (OPG) (MBS27101236), tartrate-resistant acid phosphatase 5b (MBS2702692), cross-linked N-telopeptide of type 1 collagen (NTXI) (MBS2700254), deoxypyrdinoline (DPD) (MBS2506789), osteocalcin (OC) (MBS2701838) and receptor activator of nuclear factor kappa B (RANk) (MBS2704130).

### 2.7. Histopathological Examination of Bone

The metaphysis of tibias was dissected out, fixed in 10% buffered formalin and decalcified in EDTA solution for 2 weeks. When decalcified, the specimens were embedded in paraffin. Overall, 5-µm-thick sections were then deparaffinized and stained with hematoxylin and eosin [13] and toluidine blue [14] for light microscopic examination.

### 2.8. Statistical Analysis

The data were expressed as mean ± SD. One-way analysis of variance (ANOVA) followed by Tukey’s post hoc test was done using graph pad prism. Pearson correlations were also carried out, where *p* ≤ 0.05 was considered significant.

## 3. Results

### 3.1. Effects of Orchidectomy and DHEA Treatment on Serum Levels of Testosterone and Parathyroid Hormones

**Figure 1:**
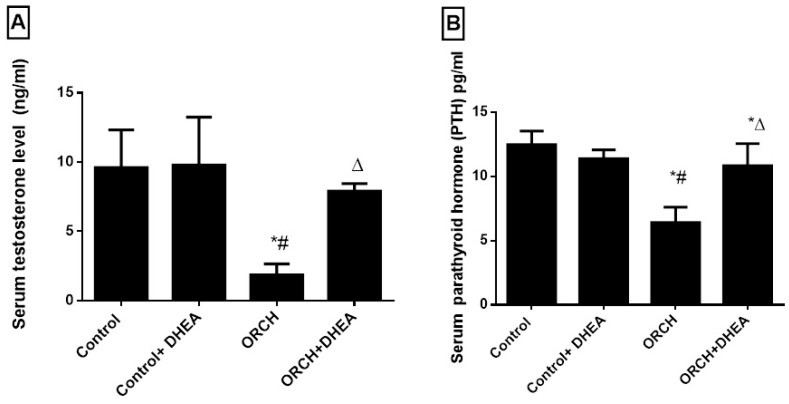
Effect of orchidectomy and DHEA (dehydroepiandrosterone) treatment on the serum levels of (**A**) testosterone and (**B**) parathyroid hormone levels. Data were expressed as mean ± SD of 8 rats. *: *p <* 0.05 versus control, #: *p <* 0.05 versus control + DHEA, ∆: *p <* 0.05 versus ORCH (orchidectomized rats).

The orchidectomized rats showed a significant (*p <* 0.0001) decrease in serum testosterone level as compared to control rats. DHEA treatment to orchidectomized rats significantly (*p <* 0.05) increased the plasma testosterone level (Figure 1A). Parathyroid hormone decreased significantly (*p <* 0.01) in response to orchidectomy. However, DHEA treatment increased this significantly (*p <* 0.05) versus the ORCH group (Figure 1B). 

### 3.2. Effect of DHEA on BMD and BMC in Orchidectomized Rats

**Figure 2:**
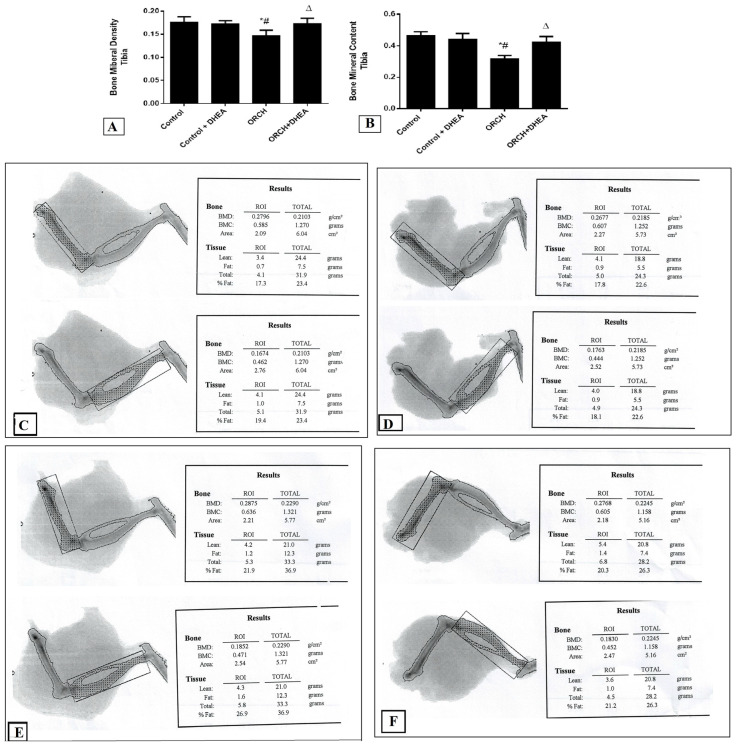
Effect of orchidectomy and DHEA treatment on (**A**) bone mineral density (BMD) and (**B**) bone mineral content (BMC) of the tibia. Representative samples of BMC and BMD from (**C**) control group, (**D**) control + DHEA, (**E**) ORCH and (**F**) ORCH + DHEA groups. Data were expressed as mean ± SD of 8 rats. *: *p* < 0.05 versus control, #: *p* < 0.05 versus control + DHEA, ∆: *p* < 0.05 versus ORCH.

Figure 2A shows that in response to DHEA in control rats the BMD of the tibia decreased insignificantly (*p* > 0.05) compared to control rats. Orchidectomy decreased the tibial BMD significantly (*p* < 0.0001) as compared to control rats and those control-treated with DHEA. On the other hand, treatment of the orchidectomized rats with DHEA increased the BMD of the tibia significantly (*p* < 0.05). Moreover, the BMC in DHEA-treated control rats decreased significantly (*p* < 0.0001) versus the control rats. Orchidectomy also decreased the BMC significantly (*p* < 0.01) as compared to control rats as well as DHEA-treated rats. DHEA treatment of the orchidectomized rats increased the BMC significantly (*p* < 0.05) versus the ORCH group but still less than the control rats (Figure 2B). Figure 2C–F are representative samples of BMC and BMD from control, control+ DHEA, ORCH and ORCH+ DHEA groups, respectively. 

### 3.3. Effect of DHEA on Osteoprotegerin (OPG) and RANK in Orchidectomized Rats

**Figure 3:**
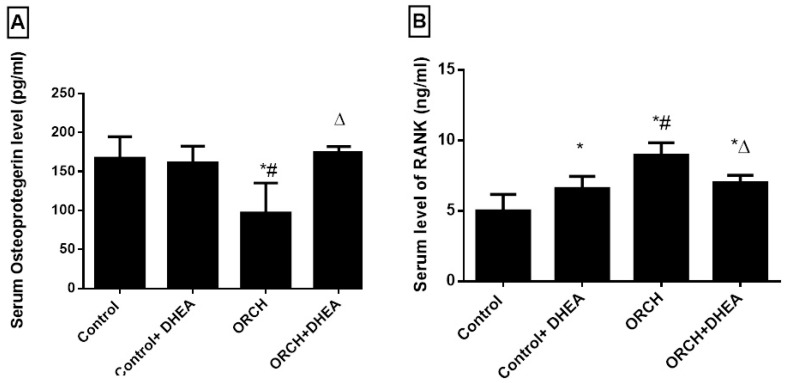
Effect of orchidectomy and DHEA treatment on serum levels of (**A**) OPG and (**B**) RANK. Data were expressed as mean ± SD of 8 rats. *: *p <* 0.05 versus control, #: *p <* 0.05 versus control + DHEA, ∆: *p <* 0.05 versus ORCH. ORCH: orchidectomy, OPG: osteoprotegrin; RANK: receptor activator of nuclear factor kappa B.

The serum level of OPG, the inhibitor of RANK, increased insignificantly (*p* > 0.05) in response to DHEA treatment to normal rats compared to control rats. Interestingly, in response to bilateral orchidectomy, OPG significantly (*p* < 0.001) decreased as compared to control rats. DHEA treatment in orchidectomized rats significantly (*p* < 0.01) increased the serum level of OPG versus the orchidectomized rats (Figure 3A). The blood level of the osteoclastogenic factor RANK increased significantly (*p* < 0.01) in orchidectomized rats as compared to control rats. DHEA treatment suppressed the upregulated RANK in orchidectomized rats significantly (*p* < 0.05) (Figure 3B).

### 3.4. Effect of DHEA on Bone Resorption Markers (Deoxypyridinoline (Dpd), Tartrate-Resistant Acid Phosphatase 5b and N-Telopeptide of Type I Collagen (NTXI)) in Orchidectomized Rats

**Figure 4:**
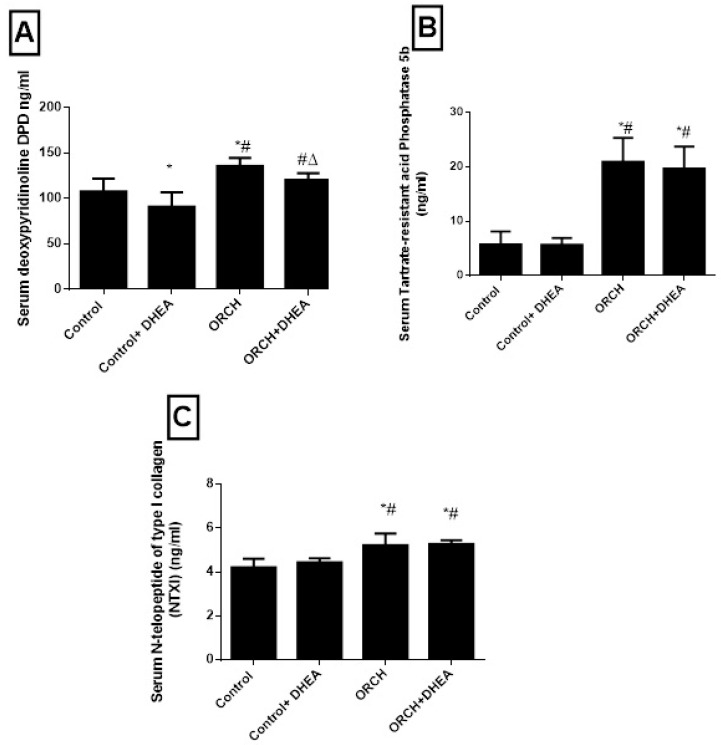
Effect of orchidectomy and DHEA treatment on serum level of on bone resorption markers (**A**) DPD, (**B**) TRAP-5b and (**C**) NTXI. Data were expressed as mean ± SD of 8 rats. *: *p <* 0.05 versus control, #: *p* < 0.05 versus control + DHEA, ∆: *p* < 0.05 versus ORCH. ORCH: orchidectomy, Dpd: deoxypyridinoline; NTXI: N-telopeptide of type I collagen; TRAP-5b: tartrate-resistant acid phosphatase 5b.

To evaluate the effect of orchidectomy on bone loss we measured the serum levels of DPD, TRAP-5b and NTXI 12 weeks after the surgery. Orchidectomy increased bone loss and raised the DPD significantly (*p* < 0.01) versus the control group (Figure 4A). Figure 4B shows that the serum level of TRAP-5b increased significantly (*p* < 0.05) in response to decreased testosterone in the orchidectomized rats as compared to control. Additionally, the serum level of NTXI increased significantly (*p* < 0.01) in orchidectomized rats as compared to control rats as shown in Figure 4C. While DHEA increased DPD in orchidectomized rats significantly (*p* < 0.05) compared to non-treated orchidectomized rats, it decreased DPD level in control rats significantly (*p* > 0.01). Furthermore, DHEA treatment increased TRAP-5b significantly (*p* < 0.001) in orchidectomized rats and insignificantly (*p* > 0.05) in control rats. The serum level of NTXI increased significantly (*p* < 0.01) in orchidectomized rats as compared to control rats as shown in Figure 4C. DHEA treatment produced an insignificant (*p* > 0.05) change of NTXI in both the control rats and orchidectomized rats as shown in Figure 4C. 

### 3.5. Effect of DHEA on Bone Formation Markers Osteocalcin (OC) and Alkaline Phosphatase (ALP)

**Figure 5:**
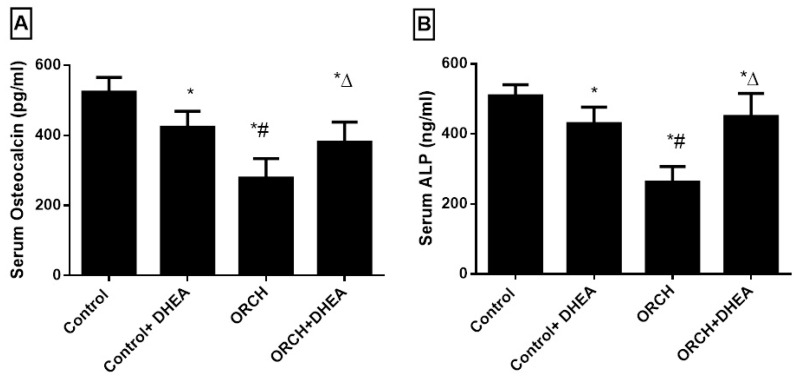
Effect of orchidectomy and DHEA treatment on serum level of bone formation markers (**A**) OC and (**B**) ALP. Data were expressed as mean ± SD of 8 rats.*: *p <* 0.05 versus control, #: *p <* 0.05 versus control + DHEA, ∆: *p <* 0.05 versus ORCH. ORCH: orchidectomy, OC: osteocalcin, ALP: alkaline phosphatase.

To evaluate the effects of DHEA on bone formation markers, we measured the serum levels of OC and ALP. Figure 5A,B shows orchidectomy decreased OC and ALP significantly (*p* < 0.05) versus the control rats. DHEA treatment in orchidectomized rats significantly increased both of them significantly versus the ORCH group.

### 3.6. Effect of DHEA on Bone Morphology in Orchidectomized Rats 

**Figure 6:**
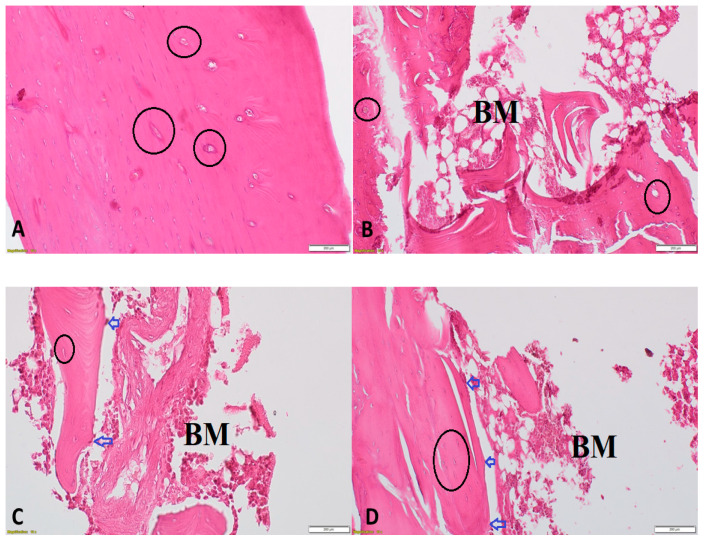
Histopathological examination using H&E of proximal metaphysis of the tibia from different groups. The specimens from control group showing a network of trabeculae of cancellous bone with osteocytes within their lacunae within bone trabeculae (black circles) (200×) (**A**), from ORCH group showing loss of trabecular bone with bone marrow spaces (BM) and few osteoblasts (blue arrows) appear rimming the endosteal surface and (200×) (**B**,**C**) and from DHEA + ORCH group showing normal trabecular bone architecture with osteoblasts at endosteal surface (200×) (**D**).

Histological changes were examined by H&E staining. In the control group, the tibiae exhibited a complete trabeculae structure and ordered arrangement of the trabeculae (Figure 6A). In the ORCH group, significantly reduced and thinning trabeculae and small numbers of empty bone lacunae were observed (Figure 6B,C). Treatment with DHEA markedly reversed these changes (Figure 6D).

### 3.7. Correlations between Serum PTH and Testosterone and Other Studied Parameters 

Serum PTH showed positive correlations with serum testosterone, OC and ALP and negative correlations with serum DPD (*p* < 0.05), while serum testosterone level showed positive correlations with BMC, BMD, serum OC, RANK and ALP and negative correlations with serum TRAP-5b (*p* < 0.05) (Table 1).

## 4. Discussion

The main findings of the present study included: a) orchidectomy in rats resulted in significant reductions in serum testosterone, PTH, BMD, BMC, OPG, osteocalcin and alkaline phosphatase (ALP) with significant increases in serum levels of RANK, DPD, TRAP-5b and NTx and b) therapy with DHEA for 12 weeks caused significant increases in serum levels of testosterone and PTH with significant improvement in BMD, BMC and markers of bone turnover. 

Previous studies examined the effect of lack testosterone in rats induced by orchidectomy on BMC and BMD. Erben et al. [16] found a high-turnover cancellous osteopenia, while Prakasam et al. [17] reported a cortical osteopenia with cortical porosity and decreased periosteal bone formation in orchidectomized rats. Iwamoto et al. [18] demonstrated that orchidectomy caused cancellous and cortical osteopenia via increasing the trabecular and endocortical bone turnover in the proximal metaphysis of the tibias. Moreover, several studies have demonstrated significant reduction in BMD in femoral and lumbar spine [19,20,21,22] 12 weeks after orchidectomy; however, others showed minimal changes in BMD [23]. In the present study, we found significant reduction in tibial BMC and BMD in orchidectomized rats 16 weeks after castration. Furthermore, histopathological examination in the current study revealed significant loss of bone mass in orchidectomized rats. Moreover, we found in the current study that DHEA hormonal replacement therapy for 12 weeks caused significant increase and improvement in tibial BMC and BMD. In line with these findings, Papierska et al. [24] demonstrated that DHEA treatment for 6–12 months caused a significant increase in bone mineral density in the lumbar spine and femoral neck. Saki et al. [19] also found that testosterone and its combination with letrozole caused significant increase in the lumbar and femoral BMD of orchidectomized rats. Moreover, Park and Omi [25] reported that DHEA treatment in ovariectomized female rats significantly improved the BMC of the lumbar spine (trabecular-abundant region). At the clinical level, Jankowski et al. [26] reported that DHEA replacement therapy at a dose of 50 mg/day for 12 months significantly improved hip BMD in older adults and spine BMD in older women.

In the current study, we found that ORCH caused significant reductions in the serum level of testosterone and DHEA replacement therapy for 12 weeks caused partial not complete correction of the serum level of testosterone in orchidectomized rats, suggesting that the biotransformation of DHEA into testosterone in rats is not complete. We reported similar findings in a previous study by our research group [27]. Arlt et al. [28] reported that DHEA is converted into estrogens by adipose and hepatic tissues, resulting in significant elevation in serum estrogen in men. Moreover, Villareal et al. [29] demonstrated that oral DHEA treatment for 6 months caused doubling of serum testosterone in women but not in men. Corona et al. [30] reported that the biological activity of DHEA is dependent on its direct biological effect and is not due to its conversion into estradiol or testosterone. Furthermore, the current study demonstrated positive correlations between serum testosterone and BMC and BMD, suggesting the involvement of testosterone in correction of osteoporosis. On the other hand, the current study showed significant reductions in serum level of PTH in orchidectomized rats and its improvement with DHEA treatment. However, we did not find any significant correlation between PTH level and BMC and BMD, suggesting that PTH might not be involved in the correction of orchidectomized-induced osteoporosis. However, serum PTH showed a negative correlation with bone resorbing biomarkers (DPD) and positive correlations with bone forming biomarkers (OC and ALP). On the other hand, Hock et al. [31] concluded that PTH treatment for 12 days caused significant increase in the bone mass in ovariectomized and orchidectomized rats and this anabolic action of PTH is not dependent on gonadal hormones. Furthermore, Tezval et al. [32] reported that PTH treatment for 5 weeks improved the bone strength in the trochanteric region of the femur.

The bone is not a static structure but consists of dynamic tissues that continuously undergo formation and breakdown. This process of continuous turnover (bone remodeling) is regulated by osteoclastic (bone eating cells) and osteoblastic (bone forming cells) cells. One of the important systems that is involved in the bone metabolism and the process of osteoclastogenesis is the RANKL/RANK/OPG. The current study demonstrated significant rise in the serum level of RANK with significant reduction in the serum level of OPG in orchidectomized rats. Furthermore, the RANK/OPG ratio was elevated in orchidectomized rats, suggesting enhancement of the processes of osteoclastogenesis and bone resorption. In line with these findings, previous studies have reported significant reduction in the serum level of OPG and the expression of OPG in rat femurs with significant increase in the serum level of RANKL and the expression of RANKL in rat femurs in ovariectomized rats [33,34,35]. However, in the current study we did not measure the serum level of RANKL, which is considered as a limitation of the current study. Moreover, in the present study, we found that DHEA therapy for 12 weeks caused significant rise in the serum level of OPG with significant decreases in the serum level of RANK, suggesting that DHEA could inhibit bone resorption and osteoclastogenesis via targeting the RANK/OPG pathway.

The markers of bone remodeling are secreted from the bone matrix or bone cells and are grouped into two categories: bone resorbing markers such as C- or N-terminal telopeptide of type I collagen (CTX or NTX) and pyridinolines that promote bone breakdown, and bone forming markers such as procollagen type I N-terminal propeptide (PINP), alkaline phosphatase (ALK), bone specific alkaline phosphatase (BS-ALP) or osteocalcin (OC) that promote bone formation. So, in the current study we investigated the effect of orchidectomy and DHEA hormonal replacement therapy on the bone resorbing biomarkers including deoxypyridinoline (Dpd), N-telopeptide of type I collagen (NTXI) and tartrate-resistant acid phosphatase 5b (TRAP-5b), and bone forming biomarkers including OC and ALP. In the current study we found significant reduction in bone forming markers including ALP and OC, suggesting reduction in bone remodeling that results in loss of bone mass. In agreement with these findings, Ryu et al. [20] found significant gradual reduction in serum levels of OC and ALP in young rats after orchidectomy. On other hand, Han and Wang, [36] reported significant elevation in bone forming biomarkers including alkaline phosphatase and bone-specific ALP and OC in ovariectomized rats.

Tartrate-resistant acid phosphatase (TRAP) is a bone resorbing biomarker that is expressed in activated macrophages, dendritic cells, and osteoclasts [37]. Serum TRAP-5b is secreted from bone-resorbing osteoclasts and its measurement reflects the presence of bone resorption in several systemic diseases that influence bone turnover [38]. Furthermore, it has been demonstrated that serum N-telopeptide of type I collagen (NTXI) is a sensitive indicator of bone resorption [39]. NTXI is excreted in urine after bone resorption and is considered as a urinary marker of bone resorption [40]. Moreover, deoxypyridinoline (DPD) is released into the blood as a result of collagen decomposition during bone turnover and is considered as a specific marker of bone resorption [41]. The current study found significant elevation in serum levels of bone resorbing biomarkers including TRAP-5b, NTX1 and DPD, suggesting enhancement of bone breakdown in orchiectomized rats as a result of testosterone deficiency. In agreement with these findings, Vanderschueren, et al. [42] reported significant elevation in serum level of DPD by 112% 66 days after orchidectomy in 12-month-old male rats and Kobayashi et al. [43] demonstrated significant elevation in serum DPD in orchidectomized dogs. Moreover, Bhardwaj et al. [44] reported a significant increase in the markers of bone resorption following ovariectomy in female rats. Moreover, DHEA treatment caused non-significant reduction in TRAP-5b and NTX1 with significant reduction in serum levels of DPD in orchidectomized rats, suggesting improvement of the process of osteogenesis.

## 5. Conclusions

We conclude that orchidectomy in male rats caused osteoporosis which was associated with significant reduction in serum levels of testosterone, PTH, OPG, OC and ALP and significant elevation in serum levels of RANK, DPD and NTx1. On the other hand, DHEA for 12 weeks corrected hypogonadism-induced osteoporosis in male rats probably via inhibiting osteoclastogenesis, stimulating the activity of osteoblasts and stimulating the secretion of PTH and testosterone.

## Figures and Tables

**Table 1:** Correlations between serum testosterone and PTH and other studied parameters.

		BMD	BMC	Serum OPG	Serum RANK	Serum DPD	Serum TRAP-5b	Serum NTX	Serum OC	Serum ALP	Serum Testost.
**Serum PTH**	r	−0.1055	0.08628	−0.04852	−0.2230	−0.4931	−0.4002	−0.09896	0.4616	0.6523	0.4950
	p	0.6079	0.6752	0.8260	0.3063	0.0022	0.0803	0.5658	0.0078	<0.0001	0.0139
**Serum Testost.**	r	0.4503	0.6955	0.3570	−0.5527	0.1078	−0.6808	0.05743	0.6500	0.6155	
	p	0.0272	0.0002	0.0945	0.0062	0.6160	0.0010	0.7898	0.0019	0.0014	

Pearson correlation, r = reflection coefficient and p = probability or significance. *p <* 0.05 was considered significant. Testost. = testosterone, ALP = alkaline phosphatase, OC = osteocalcin, AP = acid phosphatase, NTXI = N-telopeptide of type I collagen, TRAP-5b = tartrate-resistant acid phosphatase 5b, OPG = osteoprotegerin; RANK = receptor activator of nuclear factor kappa B, Dpd = deoxypyridinoline.
